# Compound heterozygous mutations in *TGFBI* cause a severe phenotype of granular corneal dystrophy type 2

**DOI:** 10.1038/s41598-021-86414-9

**Published:** 2021-03-26

**Authors:** Ikhyun Jun, Yong Woo Ji, Seung-il Choi, Bo Ram Lee, Ji Sang Min, Eung Kweon Kim

**Affiliations:** 1 Corneal Dystrophy Research Institute, Yonsei University College of Medicine, Seoul, Korea; 2 Institute of Vision Research, Department of Ophthalmology, Severance Hospital, Yonsei University College of Medicine, Seoul, Korea; 3 Saevit Eye Hospital, Goyang-Si, Gyeonggi-Do Korea

**Keywords:** Corneal diseases, Hereditary eye disease, Risk factors

## Abstract

We investigated the clinical and genetic features of patients with severe phenotype of granular corneal dystrophy type 2 (GCD2) associated with compound heterozygosity in the transforming growth factor-β-induced (*TGFBI*) gene. Patients with severe GCD2 underwent ophthalmic examination (best-corrected visual acuity test, intraocular pressure measurement, slit-lamp examination, and slit-lamp photograph analysis) and direct Sanger sequencing of whole-*TGFBI*. The patient’s family was tested to determine the pedigrees. Five novel mutations (p.(His174Asp), p.(Ile247Asn), p.(Tyr88Cys), p.(Arg257Pro), and p.(Tyr468*)) and two known mutations (p.(Asn544Ser) and p.(Arg179*)) in *TGFBI* were identified, along with p.(Arg124His), in the patients. Trans-phase of *TGFBI* second mutations was confirmed by pedigree analysis. Multiple, extensive discoid granular, and increased linear deposits were observed in the probands carrying p.(Arg124His) and other nonsense mutations. Some patients who had undergone phototherapeutic keratectomy experienced rapid recurrence (p.(Ile247Asn) and p.(Asn544Ser)); however, the cornea was well-maintained in a patient who underwent deep anterior lamellar keratoplasty (p.(Ile247Asn)). Thus, compound heterozygosity of *TGFBI* is associated with the phenotypic variability of *TGFBI* corneal dystrophies, suggesting that identifying *TGFBI* second mutations may be vital in patients with extraordinarily severe phenotypes. Our findings indicate the necessity for a more precise observation of genotype–phenotype correlation and additional care when treating *TGFBI* corneal dystrophies.

## Introduction

Several transforming growth factor beta-induced gene (*TGFBI*) mutations, with corneal deposit formation and specific clinical phenotypes, have been reported to date^1,2^. Components of *TGFBI* product, *i.e.*, transforming growth factor beta-induced protein (TGFBIp), have been detected in the deposits^3,4^, indicating a relation between *TGFBI* mutation and deposit formation. Granular corneal dystrophy type 2 (GCD2) is an autosomal dominant disorder caused by p.(Arg124His) mutation of *TGFBI*^5^. While a homozygote may show severe corneal deposits from the age of 3 years^6^, slow and progressive accumulation of granular deposits, linear deposits, and diffuse haze in the corneal stroma with aging is a characteristic pathologic feature of heterozygous GCD2^7,8^. The phenotypic variability of heterozygous GCD2 (p.(Arg124His) mutation) in the cornea is considerable, from few subtle white dots to multiple severe opacities throughout the stroma, even at relatively young ages^9,10^. However, the possible cause or mechanism of this variability has not yet been elucidated.

Severe phenotypic features due to double mutations in *TGFBI* have been reported^11–23^. In the current study, we detected five novel and two known mutations, occurring as a compound heterozygote in *TGFBI* along with p.(Arg124His), which result in a very severe phenotypic variant of GCD2. In addition, we have presented each proband’s family pedigree. Interestingly, individuals carrying a nonsense mutation in an allele and no p.(Arg124His) mutation in the opposite allele did not show an abnormal phenotype. We further performed in vitro experiments to investigate the effect of the identified mutations as well as p.(Arg124His) on TGFBIp aggregation.

## Results

### The compound heterozygous mutations p.(Arg124His) and p.(His174Asp) in *TGFBI*

**Figure 1:**
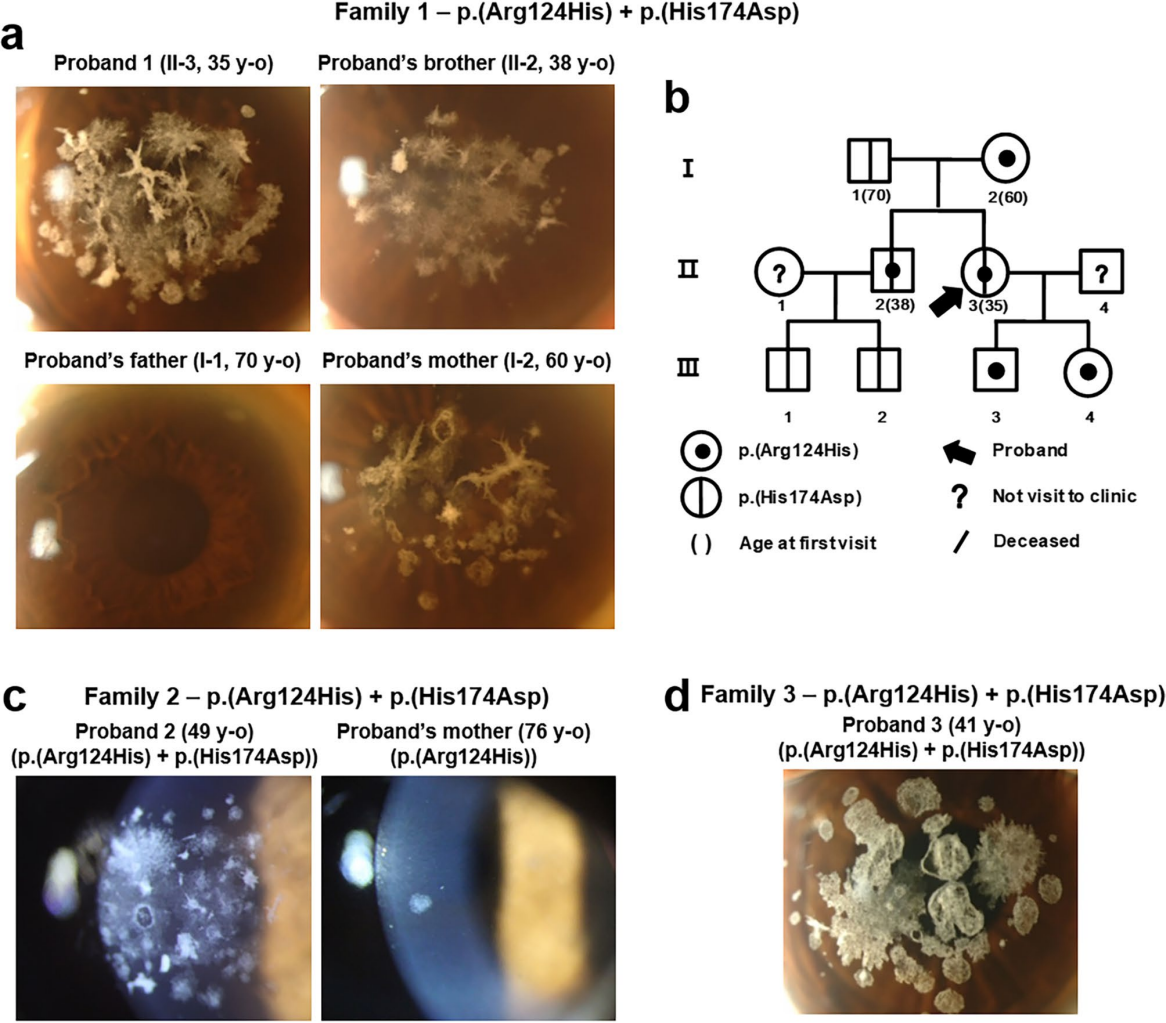
*TGFBI* p.(His174Asp) variant aggravates granular corneal dystrophy 2 caused by p.(Arg124His) mutation in a compound heterozygote. (**a**) Slit-lamp photographs of the members of Family 1. Severe confluent granular deposits with lattice deposits were observed in Proband 1. Her brother’s eyes also showed severe snowflake-like corneal deposits. (**b**) Pedigree of Family 1 showed both proband and her brother to have inherited the mutations in trans-phase. (**c**) Slit-lamp photographs of Family 2. The cornea of Proband 2 showed intensive opacities, whereas that of his mother showed mild phenotype. (**d**) Slit-lamp photograph of Proband 3 showed coarse granular and lattice deposits.

A 35-year-old woman (Proband 1; Family 1-Patient II-3) with confluent granular deposits, dense linear deposits, and extremely severe diffuse haze on her cornea visited our clinic. Her best-corrected visual acuity (BCVA) was 20/40 in both eyes (Fig. 1a). Genetic analysis revealed p.(Arg124His) and p.(His174Asp) missense mutations in *TGFBI* (Table 1). Her 38-year-old brother showed more diffuse haze in both eyes than in any usual GCD2 heterozygote of his age (Fig. 1a). The cornea of the proband’s father, who only harboured a heterozygous p.(His174Asp) mutation, was clear, whereas the mother, who was a GCD2 heterozygote, showed typical heterozygotic GCD2 phenotype, *i.e.*, annular granular deposits and deep linear deposits in the cornea. The pedigree analysis showed both the proband and her brother to have inherited the mutations in trans-phase (*i.e.*, p.(His174Asp) from the father and p.(Arg124His) from the mother) (Fig. 1b).

**Table 1:** Detected mutations increasing severity of granular corneal dystrophy 2 as a compound heterozygote with p.(Arg124His) in this study.

Family	Nucleotide change^a^	Amino acid change	Amino acid sequence conservation^b^	Frequencies in the dbSNP database^c^	Frequencies in the gnomAD database^d^	PP2^e^	MT^f^	PRO-VEAN^g^	SIFT^h^
YUGCDF-1 YUGCDF-2 YUGCDF-3	c.520C > G	p.(His174Asp)	*D. rerio*	NA	0.000012	Dam (0.999)	DC (0.999)	Del (-7.81)	Dam (0.000)
YUGCDF-4	c.740 T > A	p.(Ile247Asn)	*D. rerio*	rs376340498 C = 0.000008/1 (ExAC) A = NA	C = 0.00001444 A = NA	Dam (0.804)	DC (0.999)	Del (-5.14)	Dam (0.000)
YUGCDF-5	c.263A > G	p.(Tyr88Cys)	*D. rerio*	NA	NA	Dam (0.863)	DC (0.999)	Del (-6.93)	Dam (0.000)
YUGCDF-6	c.770G > C	p.(Arg257Pro)	*D. rerio*	rs377709778 A = 0.00003/4 (ExAC) C = NA	A = 0.00002890 C = NA	Dam (0.977)	DC (0.999)	Del (-2.86)	Dam (0.010)
YUGCDF-7	c.1631A > G	p.(Asn544Ser)	*D. rerio*	rs777288957 G = 0.00008/10 (ExAC)	0.000053	Benign (0.281)	DC (0.999)	Del (-2.97)	Dam (0.003)
YUGCDF-8	c.535C > T	p.(Arg179*)	*D. rerio*	rs886059924 (MAF = NA)	A = 0.000008133 T = NA	NA	DC (1.000)	NA	NA
YUGCDF-9	c.1404 T > A	p.(Tyr468*)	*X. tropicalis*	rs781643226 C = 0.000008/1 (ExAC) A = NA	C = 0.000004064 A = NA	NA	DC (1.000)	NA	NA

Abbreviations: Dam, damaging; DC, disease causing; Del, deleterious; MAF, minor allele frequency; MT, mutation taster; NA, not available; PP2, PolyPhen-2 prediction score Humvar; PROVEAN, Protein Variation Effect Analyzer; SIFT, Sorting Intolerant from Tolerant; SNP, single nucleotide polymorphism.

^**a**^cDNA mutations are numbered according to the human cDNA reference sequence NM_004183; + 1 corresponds to the A of ATG translation initiation codon.

^b^Amino acid residue is continually conserved throughout evolution including the species as indicated.

^c^dbSNP database (http://www.ncbi.nlm.nih.gov/SNP).

^d^gnomAD browser (http://gnomad.broadinstitute.org/).

^e^PolyPhen-2 prediction score HumVar ranges from 0 to 1.0; 0 = benign, 1.0 = probably damaging (http://genetics.bwh.harvard.edu/pph2/).

^f^Mutation taster (http://www.mutationtaster.org/). ^g^PROVEAN (http://provean.jcvi.org/index.php). ^h^SIFT (http://sift.jcvi.org/).

A 49-year-old man (Proband 2; Family 2-Patient II-2), not related to Family 1, visited our clinic with visual disturbances complaint; he presented with very severe diffuse haze and stromal opacities compared to other patients (Fig. 1c). Genetic and segregation analyses of the patient’s family showed compound heterozygous p.(Arg124His) and p.(His174Asp) mutations in *TGFBI* (Supplementary Fig. S1a).

A 41-year-old woman (Proband 3; Family 3), not related to either Family 1 or 2, presented with severe phenotypic variation (Fig. 1d) and showed both p.(His174Asp) and p.(Arg124His) mutations in *TGFBI* upon genetic analysis. We, however, could not trace her pedigree, since the patient refused clinical evaluation and genetic analysis of her family members.

To investigate the molecular mechanism of why the phenotype of GCD2 aggravate when p.(His174Asp) mutation is accompanied, the degree of aggregation of p.(His174Asp), and p.(Arg124His) mutant proteins was examined. Mixture of the cultured media of p.(His174Asp) expressing cells and p.(Arg124His) corneal fibroblasts, showed increased TGFBIp oligomer and aggregate formation during in vitro experiments (Supplementary Fig. S2, see also Supplementary Note).

### Compound heterozygous mutations, p.(Arg124His) and p.(Ile247Asn), in *TGFBI*

**Figure 2:**
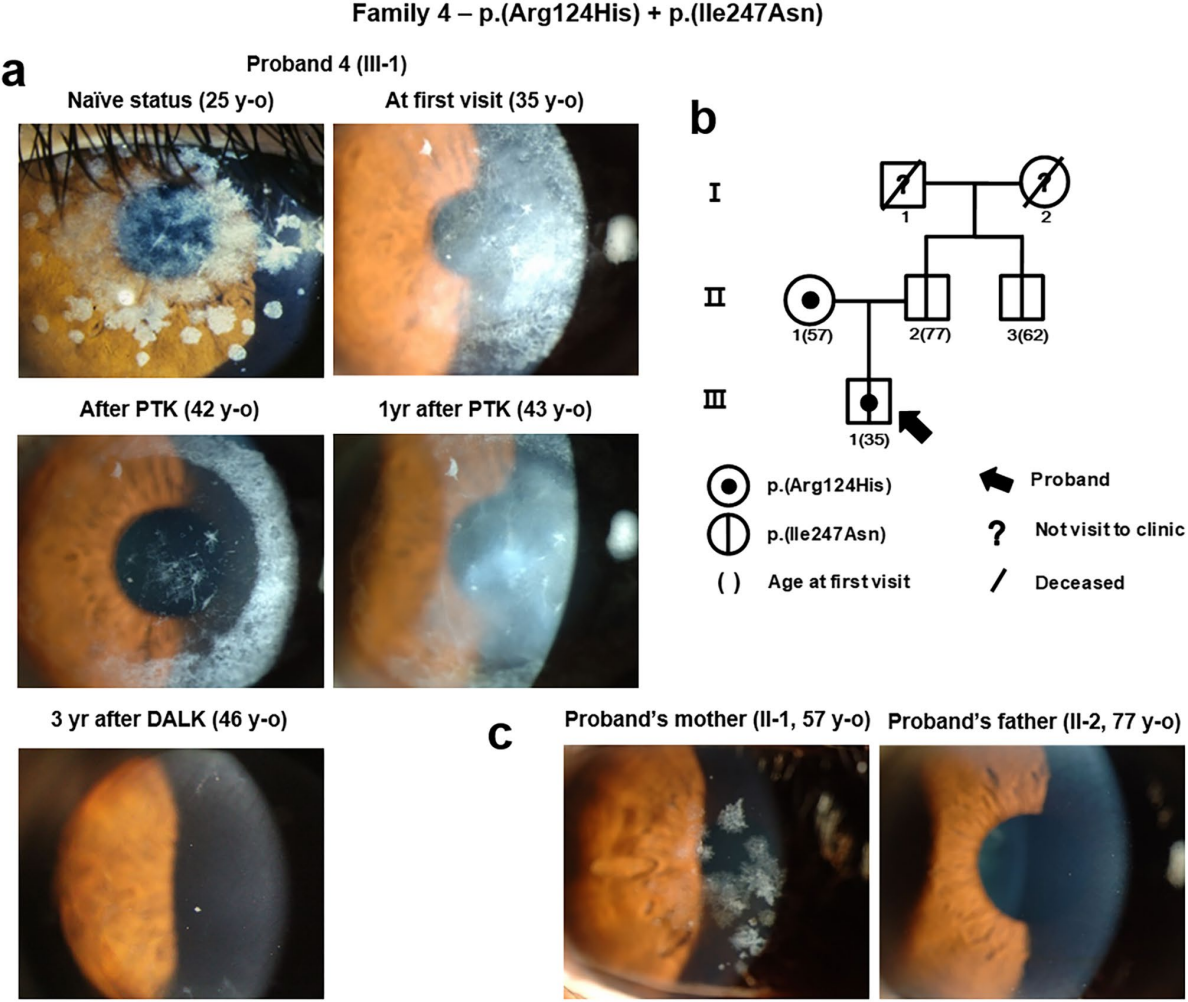
Compound heterozygous mutations, p.(Ile247Asn) and p.(Arg124His), in *TGFBI*. (**a**) Slit-lamp photographs of Proband 4 (Family 4). Intensive snowflake-like corneal deposits were noted at the age of 25 years, and rapid recurrence occurred after phototherapeutic keratectomy (PTK). Following deep anterior lamellar keratoplasty (DALK), the cornea has been well-maintained since the past 3 years. (**b**) Pedigree of Family 4 confirmed compound heterozygosity. (**c**) Slit-lamp photographs of the parents of Proband 4. The mother, who only had heterozygous p.(Arg124His) mutation in *TGFBI*, showed milder phenotypes than the son, and the father, who had heterozygous p.(Ile247Asn) mutation in *TGFBI*, showed a clear cornea. Although *TGFBI* p.(Ile247Asn) mutation itself was silent, when accompanying p.(Arg124His) mutation, phenotypes of granular corneal dystrophy 2 became extensive and recurrence occurred rapidly after PTK.

A 35-year-old man (Proband 4; Family 4-Patient III-1) was referred to our hospital with severe diffuse corneal haze causing visual disturbance, which recurred after phototherapeutic keratectomy (PTK) at another clinic. We obtained photographs of the cornea, acquired at the other clinic at the age of 25 years; he had undergone PTK of the right cornea at the age of 24 years, while the left cornea had not been treated. The photograph of his left untreated cornea acquired at the age of 25 years (Fig. 2a left first) showed extremely severe diffuse haze and granular deposits. Both p.(Ile247Asn) and p.(Arg124His) missense mutations were detected in *TGFBI* (Table 1), and pedigree analysis confirmed the mutations to be located in different alleles (Fig. 2b). The proband’s father, aged 77 years, showed clear corneas (Fig. 2c) with only p.(Ile247Asn) mutation, whereas the proband’s mother harboured p.(Arg124His) mutation and her cornea showed typical GCD2 features. Moreover, the 62-year-old paternal uncle of the proband harboured only p.(Ile247Asn) mutation with a clear cornea. Since the proband experienced decreased visual acuity, additional PTK for each eye was performed separately in our clinic. Corneal opacity of the proband, however, recurred rapidly, becoming diffuse and dense even after several PTK ablations. After deep anterior lamellar keratoplasty (DALK) of his left eye, the cornea has remained clear since the past 3 years (Fig. 2a).

Similar to p.(His174Asp), the p.(Ile24yAsn) mutation was also examined for the aggregation of TGFBIp. Mixture of the cultured media of p.(Ile247Asn) expressing cells and p.(Arg124His) corneal fibroblasts, showed increased TGFBIp oligomer and aggregate formation during in vitro experiments (Supplementary Fig. S2, see also Supplementary Note).

### Heterozygous patients with GCD2 having additional p.(Tyr88Cys), p.(Arg257Pro), or p.(Asn544Ser) mutation in a different *TGFBI* allele

**Figure 3:**
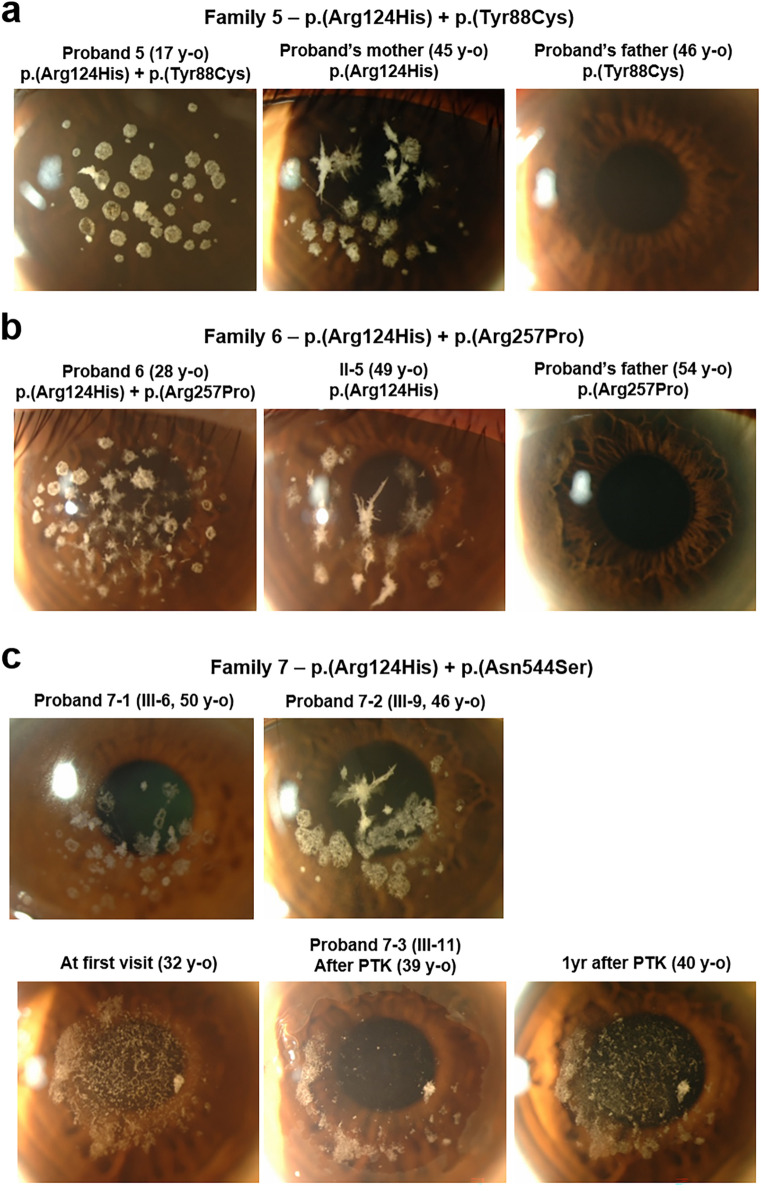
*TGFBI* p.(Tyr88Cys), p.(Arg257Pro), and p.(Asn544Ser) mutations were accompanied by p.(Arg124His) mutation in patients with severe phenotypes. (**a**) Slit-lamp photographs of Proband 5 and her parents (Family 5). The proband showed numerous large discoid granular deposits. However, her mother carrying heterozygous p.(Arg124His) variant showed age-appropriate disease pattern, while the father carrying heterozygous p.(Tyr88Cys) variant showed no disease phenotype. (**b**) Slit-lamp photographs of the members of Family 6. Compound heterozygosity of *TGFBI* p.(Arg124His) and p.(Arg257Pro) mutations caused severe GCD2 phenotypes compared to the heterozygosity of *TGFBI* p.(Arg124His) mutation alone, although heterozygosity of *TGFBI* p.(Arg257Pro) mutation did not cause disease. (**c**) Slit-lamp photographs of Proband 7 carrying compound heterozygous mutations of *TGFBI* (p.(Arg124His) and p.(Asn544Ser)). The recurrence was very rapid after phototherapeutic keratectomy (PTK).

A 17-year-old woman (Proband 5; Family 5-Patient II-2) with numerous large granular deposits in the cornea visited our clinic. She experienced intermittent eye pain due to recurrent corneal erosion, although her BCVA was 20/25 in both eyes (Fig. 3a). Whole-*TGFBI* sequencing and pedigree analysis of the patient revealed that she harboured compound heterozygous p.(Arg124His) and p.(Tyr88Cys) mutations (Table 1, Supplementary Fig. S1b). No corneal deposit was detected in her 46-year-old father who harboured p.(Tyr88Cys) heterozygous mutation alone, whereas her 45-year-old mother harbouring p.(Arg124His) heterozygous mutation showed typical GCD2 heterozygous phenotype with discoid granular and star-shaped corneal deposits in both eyes (Fig. 3a). The proband’s sibling, with no mutation, showed no corneal opacity.

A 28-year-old man (Proband 6; Family 6-Patient III-1) was referred to our clinic owing to visual disturbance due to corneal opacities in both eyes. BCVA of the patient was 20/40 for the right eye and 20/35 for the left eye, with both eyes showing confluent granular deposits and dense lattice deposits (Fig. 3b). Genetic analysis of the proband showed p.(Arg124His) and p.(Arg257Pro) missense mutations in *TGFBI* (Table 1, Supplementary Fig. S1c). His 54-year-old father, who only harboured heterozygous p.(Arg257Pro) mutation, did not show any deposit (Fig. 3b), whereas his 51-year-old mother and 49-year-old maternal uncle, both GCD2 heterozygotes, showed typical annular granular deposits with deep linear deposits (Fig. 3b), confirming trans-phase mutations in the proband.

A 32-year-old woman (Proband 7; Family 7-Patient III-11), who had undergone laser-assisted sub-epithelial keratomileusis (LASEK) in both her eyes at another clinic, visited our clinic with complains of visual disturbance, with severe diffuse stromal opacities. She underwent PTK in our clinic to remove the opacity in her right eye; however, her corneal opacity recurred rapidly after PTK (Fig. 3c). Genetic and pedigree analyses revealed that she carried both p.(Arg124His) and p.(Asn544Ser) mutations in a different allele of *TGFBI* (Table 1, Supplementary Fig. S1d). We could not find older living family members with p.(Asn544Ser) mutation alone in her family, and the younger members (aged 15 and 4 years) who harboured p.(Asn544Ser) mutation alone did not show any opacity.

We happened to identify a 22-year-old woman with p.(Asn544Ser) mutation alone in a different family during routine genetic screening before refractive surgery, following which we analysed the whole family of the patient for p.(Asn544Ser) mutation (Supplementary Fig. S1g). Two family members, one 82-year-old woman and the other 74-year-old woman, who were p.(Asn544Ser) heterozygotes, did not show any corneal opacity. A 56-year-old woman with p.(Asn544Ser) mutation alone (Family 10-Patient III-5), who had undergone LASEK 4 years ago on her left cornea only, to correct myopia of 2.5 D, showed single, small, white opacity, 1.4 mm away from the pupil centre (Supplementary Fig. S1h).

### Heterozygous patients with GCD2 and additional nonsense mutations (p.(Arg179*) or p.(Tyr468*)) in the opposite allele of *TGFBI*

**Figure 4:**
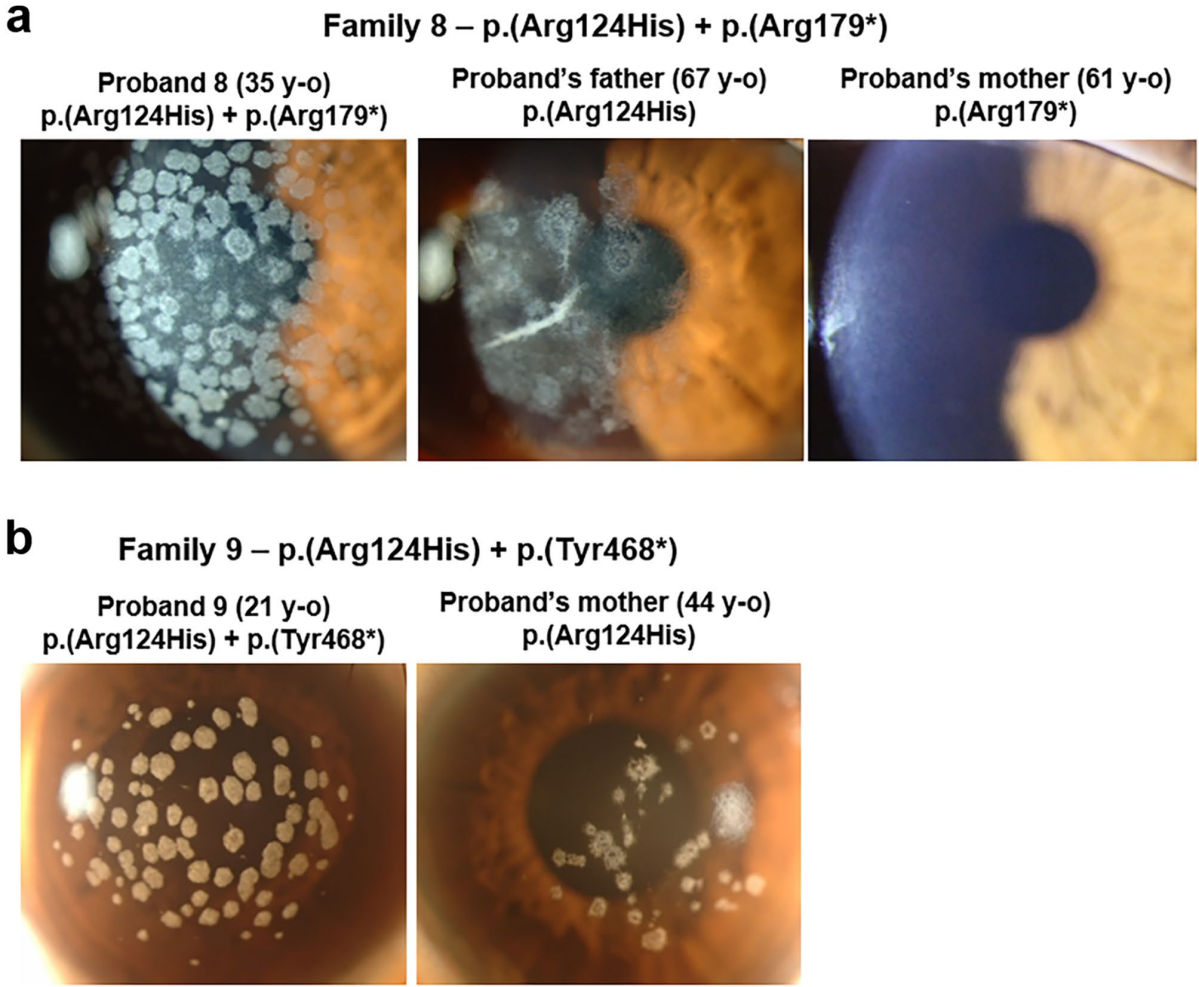
Nonsense mutations of *TGFBI* (p.(Arg179*) and p.(Tyr468*)) manifested severe granular corneal dystrophy 2 (GCD2) phenotypes. (**a**) Slit-lamp photographs of members of Family 8. Extensive multiple discoid granular deposits, with some annular deposits, were detected, resembling the features of homozygous GCD2 in Proband 8 carrying *TGFBI* p.(Arg179*) and p.(Arg124His) mutations. Her father carrying heterozygous *TGFBI* p.(Arg124His) mutation showed milder phenotypes than the proband, and the mother carrying heterozygous *TGFBI* p.(Arg179*) showed no corneal opacity. (**b**) Slit-lamp photographs of Proband 9 and his mother. Proband 9 harboured compound heterozygous *TGFBI* p.(Tyr468*) and p.(Arg124His) mutations and showed more severe discoid granular deposits than his mother who harboured heterozygous *TGFBI* p.(Arg124His) mutation.

A 35-year-old woman (Proband 8; Family 8-Patient III-2) visited our clinic for treating extensive severe multiple discoid granular corneal deposits, resembling those observed in homozygous GCD2, and diffuse haze in both eyes. She had BCVA of 20/50 in both eyes and no other ocular history (Fig. 4a). After whole-*TGFBI* sequencing, she was found to carry compound heterozygous p.(Arg124His) and p.(Arg179*) [CGA (Arg) → TGA (stop codon)] mutations (Table 1). The proband’s 76-year-old father and 36-year-old elder sister were found to be p.(Arg124His) heterozygotes with their corneas expressing the typical GCD2 phenotypes (father: superficial breadcrumb-like deposits with deep spiny deposits) (Fig. 4a). The younger sister, without mutation, had no corneal abnormality. The proband’s mother and two maternal aunts, who were 70-, 64-, and 56-year-old, respectively, showed clear corneas despite having a missense mutation (p.(Arg179*)) in *TGFBI* (Fig. 4a, and Supplementary Fig. S1e). The findings for this family have been previously reported^21^, except for the results of the genetic tests of the proband’s mother and maternal aunts.

A 21-year-old man (Proband 9; Family 9-Patient II-1) with numerous large granular deposits in both eyes visited our clinic (Fig. 4b). Both p.(Arg124His) and p.(Tyr468*) [TAT (Tyr) → TAA (stop codon)] mutations were detected in *TGFBI* (Table 1). The proband’s 44-year-old mother harbouring p.(Arg124His) mutation showed only mild typical corneal opacities compared to the proband (Fig. 4b). Although we could not test whether the his father harboured p.(Tyr468*) mutation, the proband was likely compound heterozygous for these mutations, assuming that p.(Tyr468*) mutation did not occur de novo (Supplementary Fig. S1f.).

## Discussion

The current study demonstrated that simultaneous presence of mutations, such as p.(His174Asp), p.(Ile247Asn), p.(Tyr88Cys), p.(Arg257Pro), p.(Asn544Ser), p.(Arg179*), and p.(Tyr468*), along with p.(Arg124His), in *TGFBI* in a compound heterozygous pattern could result in the severe phenotype of GCD2. p.(Tyr88Cys), p.(Arg179*), or p.(Tyr468*) mutation showed increased number or size of granules, while the other mutations showed increased amounts of linear deposits. Compound mutations of p.(Arg124His) with p.(Asn544Ser) or p.(Arg179*) have been reported previously, without identifying the phenotype of family members having p.(Asn544Ser) or p.(Arg179*) mutation alone^16,21^; such aspects have been presented herein. The remaining five variants are newly detected mutations.

Double mutations with p.(Arg124His) in *TGFBI* have been identified in only 3 cases to date^16,20,21^, out of 12 *TGFBI* double mutations reported in 13 previous studies (Supplementary Table S2)^11–23^. Whether the previously reported p.(Ser104Lysfs*27) and p.(Arg179*) mutations carrying p.(Arg124His)^20,21^ were compound heterozygous, could not be confirmed because of the refusal of family members for evaluation. We found five novel and two known *TGFBI* variants in compound heterozygotes with p.(Arg124His) and verified that each of the second mutation identified caused no corneal abnormality when present by itself. These results had significant clinical implications, since unexpected severe p.(Arg124His) variant can arise in families when p.(Arg124His) mutation is combined with the silent mutations mentioned above.

The cause of the presentation of the severe phenotype of GCD2 in individuals with compound heterozygous mutations of *TGFBI* is not yet clear. Mixture of TGFBIps obtained from the culture media of cells expressing p.(His174Asp)- or p.(Ile247Asn), and p.(Arg124His)-mutant corneal fibroblasts, showed increased oligomer and aggregate formation in in vitro experiments. Several studies have reported that homozygous GCD2 corneal fibroblasts showed more oxidative stress and impaired autophagy functions, resulting in a greater accumulation of TGFBIp in the corneal fibroblasts^24,25^. Further, intracellular TGFBIp is reportedly cleared out via lysosomes, and this function is impaired in cultured GCD2 corneal fibroblasts^26^. We suspected the double production of mutated TGFBIps from p.(Arg124His) allele and heterozygous mutation in opposite alleles to result in an intracellular TGFBIp status closer to that seen in GCD2 homozygotes than in heterozygotes. Elucidating the mechanism underlying the formation of differential phenotypes for each different second mutation requires further studies.

Interestingly, all the heterozygotes of p.(His174Asp) (70-year-old), p.(Ile247Asn) (77- and 62-year-old), p.(Tyr88Cys) (46-year-old), p.(Arg257Pro) (64- and 54-year-old), and p.(Arg179*) (70-, 64-, 61-, and 56-year-old) mutations showed clear corneas even at advanced age. Moreover, p.(Asn544Ser) heterozygotes, both 82- and 74-year-old Korean women with the mutation, showed clear cornea (Supplementary Fig. S1h). The 56-year-old woman with p.(Asn544Ser) mutation alone, who had undergone LASEK 4 years ago, showed only a small opacity. We could not determine whether this opacity was a result of scarring after LASEK or the exacerbation of silent cornea having p.(Asn544Ser)-only mutation. A study conducted in Japan reported lattice corneal dystrophy in a 68-year-old patient with a p.(Asn544Ser) mutation only^27^, although the underlying cause could not be precisely explained. However, the data suggested that more reports of cases with silent second mutations would be required to verify the safety of refractive surgery. There are abundant reports of exacerbation of *TGFBI*-related corneal dystrophies, including GCD2 or GCD1 with deposits, following corneal trauma such as laser-assisted in situ keratomileusis^28,29^, LASEK, photorefractive keratectomy, and refractive keratotomy^4,30,31^.

Two types of mutations with stop codons, p.(Arg179*) or p.(Tyr468*), combined with p.(Arg124His) mutation in *TGFBI* showed similar phenotypes in our study, with extensive discoid granular deposits resembling those in homozygous GCD2. Yam et al*.* reported a 31-year-old patient carrying compound heterozygous p.(Arg124His) and p.(Leu103Leufs*28) mutations (c.307_308delCT induced frame shift and finally resulted in premature stop at position 130) in *TGFBI*, showing a pattern of corneal opacities very similar to that in our cases^20^. In the present study, p.(Arg179*) or p.(Tyr468*) stop codon was generated at codon 179 or 468, while in the study conducted by Yam et al., the stop codon occurred with a frame shift; however, the phenotype remained very similar. These data collectively showed that the presence of compound heterozygous mutation with the stop codon in one allele and p.(Arg124His) mutation in another could result in extensive granular deposits. Poulsen et al*.*^32^ reported that the macroscopic, microscopic, and ultrastructural appearance of *TGFBI*-null mouse cornea remain unaffected, suggesting that partial or complete knockdown of *TGFBI* could be a potential therapy against *TGFBI*-linked corneal dystrophies. Currently, correction of p.(Arg124His) mutation in cornea by knocking out the mutated allele is being attempted for the treatment of GCD2^33,34^. Our present data indicate that the knockout of the allele should be very precise during gene therapy, in case of the heterozygote, such that only the allele containing p.(Arg124His) mutation is treated, leaving the normal-sequence allele intact. If the wild-type allele is altered to stop codon while the p.(Arg124His)-mutant allele is kept unchanged, it may result in the final corneal lesion being similar to that observed in compound heterozygotes with stop codon in the present study.

Family 1 in this study was previously reported to have extremely varied GCD2 heterozygote phenotypes^10^. However, the exact reason for severe phenotypic variability in the family was not known then; here, we showed the compound heterozygous mutation to be a causative factor among multiple genetic and environmental factors. Moreover, we showed that the patients with compound heterozygous *TGFBI* mutations experienced rapid recurrence after PTK. Identifying the reason underlying why the cornea remaining clear for an extended period after DALK in Proband 4 of this study would require further investigation.

In conclusion, this study reported five novel and two known genetic mutations that can predict the occurrence of the severe phenotype of heterozygous GCD2. Since phenotypic variations may indicate the presence of a new genetic change, whole-*TGFBI* sequencing would be necessary when a heterozygous patient with severe GCD2 is identified. Identification of cases with second mutations could more thoroughly explain the pathophysiology of GCD2.

## Methods

### Clinical investigation

This study was approved by the Institutional Review Board of Yonsei University College of Medicine (IRB No. 4–2012-0209) and followed the tenets of the Declaration of Helsinki. Written informed consent was obtained from all participants. All participants agreed to have their photos published. The patients who visited Severance Hospital from January 2007 to October 2020 underwent a detailed ophthalmological examination, including BCVA test, intraocular pressure measurement, slit-lamp examination, and slit-lamp photograph analysis. Among the patients with GCD2, the ones who presented severe phenotypes and had no other history of ocular or systemic diseases were selected as candidates for whole-*TGFBI* sequencing. After identifying other mutations, in addition to p.(Arg124His), family members were enrolled in the study to perform segregation analysis.

### Genomic DNA preparation and mutation analysis

*TGFBI* was analysed as described previously, after receiving informed consent from the participants^29^. Briefly, 2 ml of blood was drawn from each subject and genomic DNA was extracted from their peripheral leukocytes using a QIAamp DNA Blood Mini Kit (Qiagen, Hilden, Germany) according to the manufacturer’s instructions. Primers were designed to amplify all 17 exons of *TGFBI* (Supplementary Table S1). Each polymerase chain reaction (PCR) was performed using a 20-μl reaction mixture (Maxime PCR Premix kit; iNtRON Biotechnology, Seongnam-si, Gyeonggi-do, Korea) containing 100 ng of genomic DNA, 10 pmol each of forward and reverse primers, and distilled water. Samples were amplified through 35 PCR cycles; each cycle consisted of denaturation for 20 s at 94 °C, annealing for 15 s at 58 °C, and extension for 50 s at 72 °C. PCR was performed using a 96-well thermal cycler (Applied Biosystems, Foster City, CA, USA). Subsequently, Sanger sequencing was performed for all exons; to screen for mutations, we compared the DNA sequence of patients with the complementary DNA sequence of *TGFBI* obtained from GenBank (NC_000005.10).

### TGFBIp oligomer formation, aggregate analysis, and immunoblotting

To determine oligomer formation and aggregation ability, both wild-type and p.Arg124His-mutant TGFBIp were incubated with either p.(His174Asp)- or p.(Ile247Asn)-mutant TGFBIp for 1 h at 37 °C, following which immunoblotting was performed^35^. Details are available in the Supplementary Methods.

## Supplementary Information

Supplementary Information
