# Supplementary material for: Compound heterozygous mutations in TGFBI cause a severe phenotype of granular corneal dystrophy type 2
Source: Sci Rep. 2021 Mar 26;11:6986. doi: 10.1038/s41598-021-86414-9 (PMC7997986; doi:10.1038/s41598-021-86414-9)
Supplement: Supplementary file 1 — Supplementary Information [file 41598_2021_86414_MOESM1_ESM.pdf]

Compound Heterozygous Mutations in *TGFBI* Cause a Severe Phenotype of Granular Corneal Dystrophy Type 2

Ikhyun Jun[1,2], Yong Woo Ji[2], Seung-il Choi[1], Bo Ram Lee[1], Ji Sang Min[2], Eung Kweon Kim[1,2,3]

[1] Corneal Dystrophy Research Institute, Yonsei University College of Medicine, Seoul, Korea

[2] Institute of Vision Research, Department of Ophthalmology, Severance Hospital, Yonsei University College of Medicine, Seoul, Korea

[3]Department of Ophthalmology, Saevit Eye Hospital, Goyang, Korea

Supplementary Methods

Cell culture, plasmids, and antibodies

Human embryonic kidney 293 (HEK293) cells were cultured in Dulbecco's modified Eagle medium (DMEM) (Corning Cellgro, Manassas, VA, USA) supplemented with 10% (v/v) fetal bovine serum (FBS), 100 U/mL penicillin, and 0.1 mg/mL streptomycin. Normal and GCD2 (TGFBI p.(Arg124His) mutant) homozygous primary corneal fibroblasts were prepared using previously described methods.[29] The cDNA of *TGFBI* was generated by RT-PCR from mRNA obtained from human corneal fibroblasts. The cDNA of *TGFBI* was subcloned into pcDNA3.1 vector with C-terminal V5-tag using PCR amplification. The TGFBI p.(His174Asp) and p.(Ile247Asn) mutant plasmids were generated using PCR-based site-directed mutagenesis. Plasmids were transiently transfected into HEK293 cells using Lipofectamine 2000 (Invitrogen Carlsbad, CA).

TGFBI Oligomer and Aggregates Analysis and Immunoblotting

After wild type (WT) and GCD2 homozygous primary corneal fibroblasts reached 90% confluence, the culturing media were changed to DMEM without FBS and the cells were cultured for 5 days. The media were switched serum free condition during the time of TGFBIp collection to be free from the influence of serum component which influences the Western blot data. The serum free media of WT and p.(Arg124His) mutant cultured cells were collected. Then the cultured media was collected. The plasmids expressing TGFBI p.(His174Asp) and p.(Ile247Asn) were transfected into HEK293 cells. Transfected HEK293 cells were cultured for 3 days after changing the media to DMEM without FBS. The p.(His174Asp) and p.(Ile247Asn) mutated TGFBIp containing media was collected after confirmation of mutated TGFBIp overexpression by Western blot analysis of cell lysates and

media. The collected media was mixed and incubated for 1 h at 37℃. Then the media samples were suspended in a sodium dodecyl sulfate buffer with or without β-mercaptoethanol, and separated by sodium dodecyl sulfate-polyacrylamide gel electrophoresis. The separated proteins were transferred to a nitrocellulose membrane and blotted with the appropriate primary and secondary antibodies. The anti-TGFBI (R&D Systems, Minneapolis, MN) antibody was used as the primary antibody, and an anti-goat IgG (HRP) (Amersham Pharmacia Biotechnology, Piscataway, NJ) antibody was used as the secondary antibody. Protein bands were detected by enhanced chemiluminescence (Thermo Scientific).

Supplementary Note

p.(His174Asp) and p.(Ile247Asn) TGFBIp enhanced TGFBIp Aggregation

We investigated the amyloidogenic properties of the commonly occurring mutant p.(Arg124His)-TGFBIp in combination with p.(His174Asp) or p.(Ile247Asn) mutant (Supplementary Figure 2). WT and p.(Arg124His) TGFBIp secreted from corneal fibroblasts were incubated with or without p.(His174Asp) or p.(Ile247Asn) secreted from transfected HEK293 cells for 1h at room temperature. Oligomeric TGFBIp was significantly increased in p.(Arg124His)-TGFBIp than WT. Although it cannot reach statistical significance, there is a tendency that p.(Arg124His) was more oligomerized with p.(His174Asp) or p.(Ile247Asn)-TGFBIp than WT. Furthermore, when immunoblot analysis were performed presence of reducing agents (2.5% β-mercaptoethanol), aggregates of TGFBIp were only detected in columns containing p.(Arg124His)-TGFBIp, and it was increased in the presence of p.(His174Asp) and p.(Ile247Asn)-TGFBIp.

Supplementary Figures

Supplementary Figure S1.

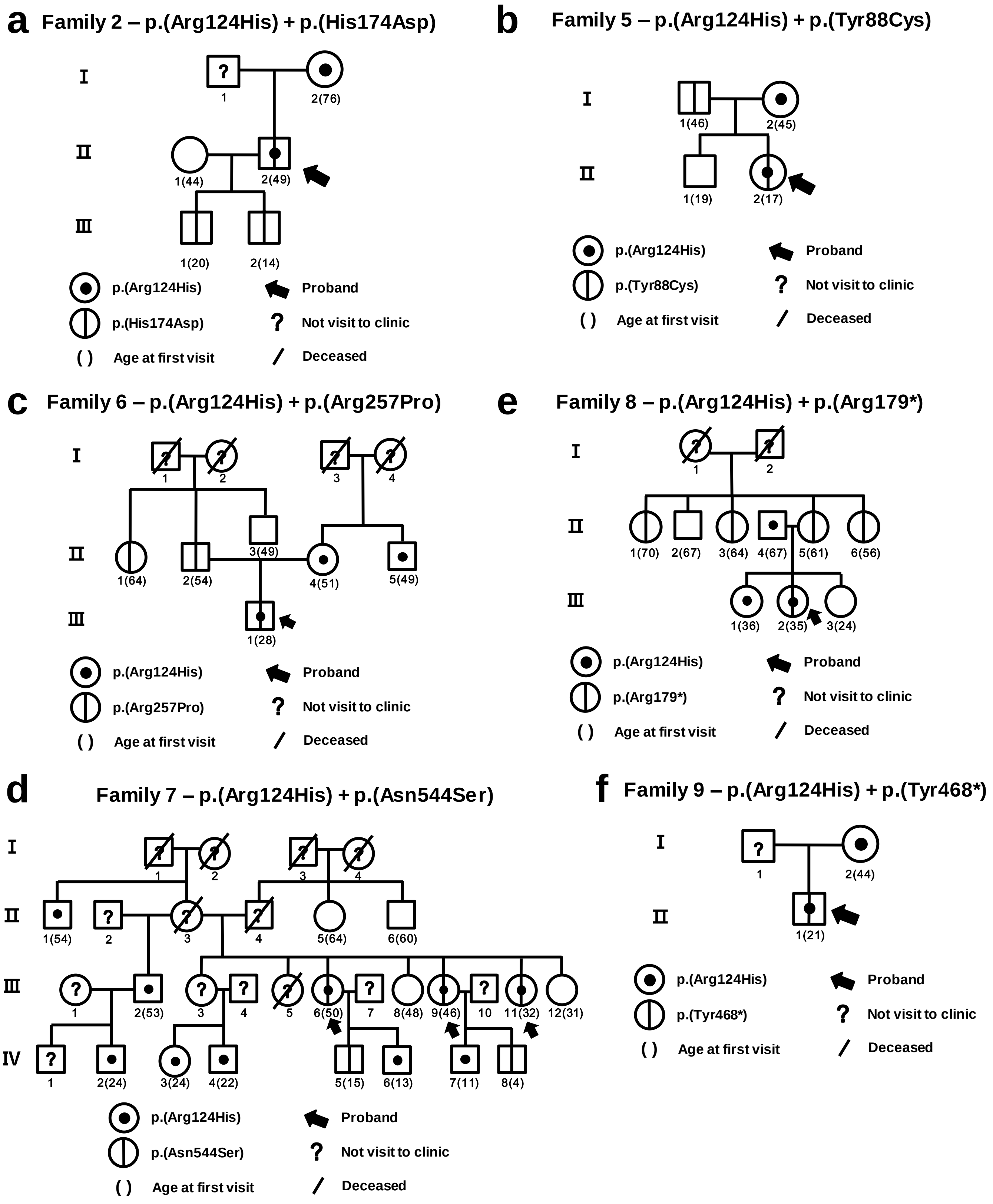

g

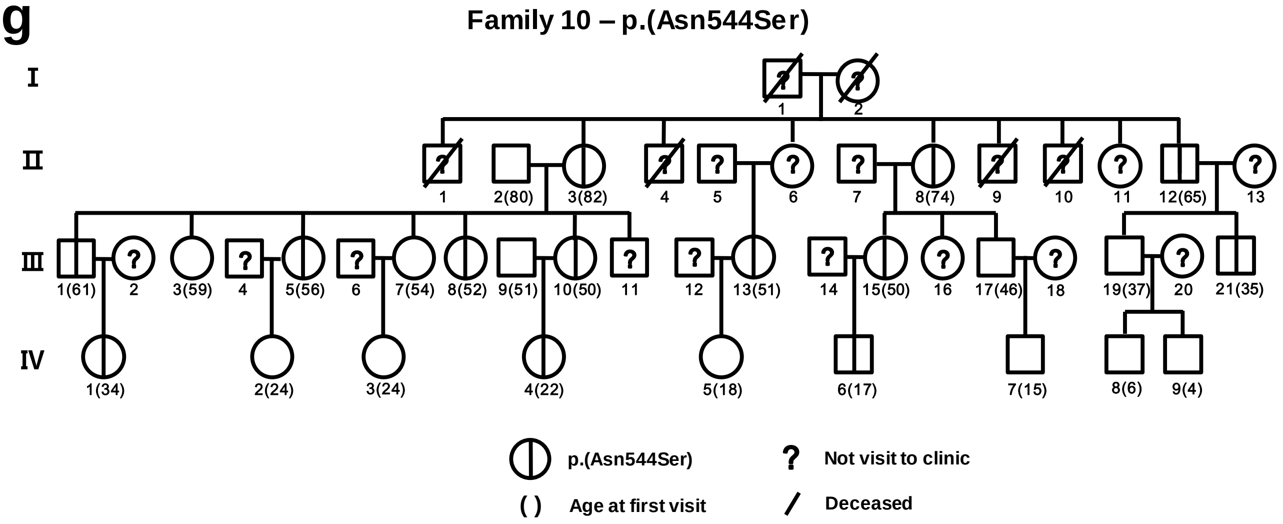

h

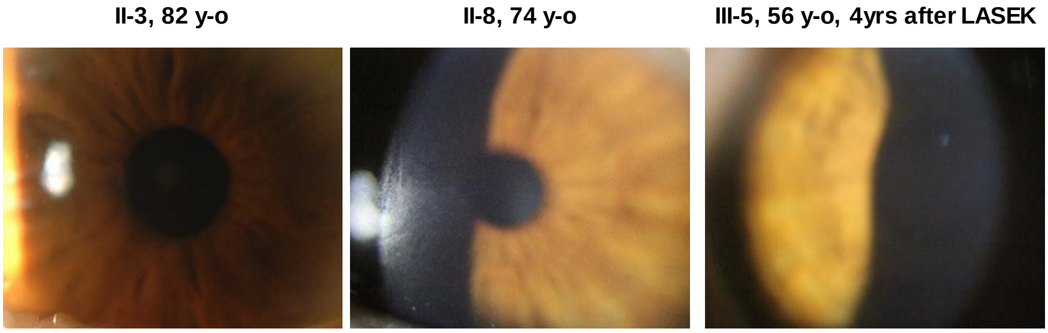

Supplementary Figure S1. Pedigree analysis of Family 2 (A), 5 (B), 6 (C), 7 (D), 8 (E), and 9 (F). All detected variants in this study were located in opposite allele to p.(Arg124His) which can cause granular dystrophy 2. (G) Pedigree analysis of Family 10 (*TGFBI* p.(Asn544Ser) only). (H) Slit-lamp photographs of the family members carrying *TGFBI* p.(Asn544Ser) only. The p.(Asn544Ser) only subjects (II-3;82 year-old, and II-8; 74 year-old) showed clear cornea. The 56-year-old woman with p.(Asn544Ser) only, who received LASEK showed only single small white opacity.

Supplementary Figure S2.

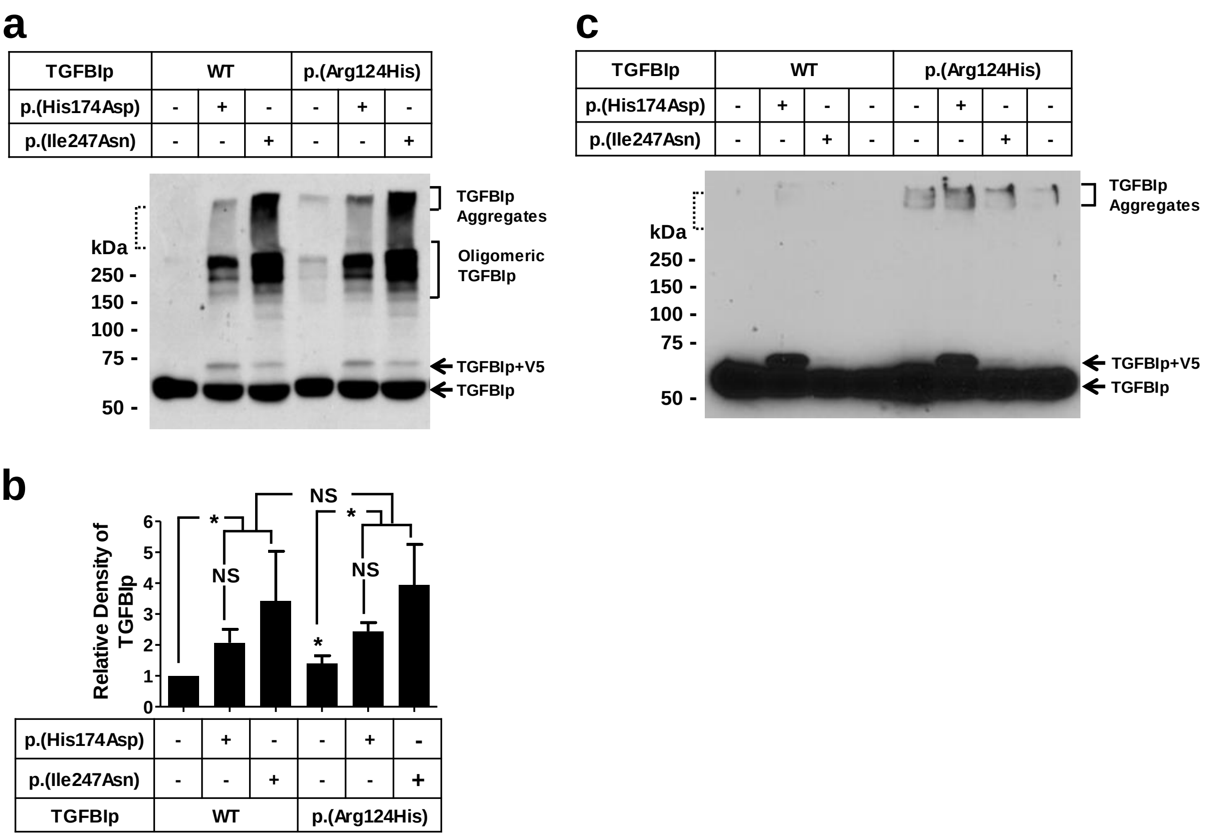

Supplementary Figure S2. TGFBIp p.(His174Asp) and p.(Ile247Asn), which made no disorder alone, increased oligomerization and aggregates in combination with TGFBIp p.(Arg124His). (A) Immunoblot analysis of TGFBIp aggregation and oligomerization generated in presence or absence of mutant-TGFBIp. TGFBIp samples were incubated for 1 h at 37°C then immunoblotted without β-mercaptoethanol. Lane 1: WT; lane 2: WT + p.(His174Asp); lane 3: WT + p.(Ile247Asn); lane 4: p.(Arg124His); lane 5: p.(Arg124His) + p.(His174Asp); lane 6: p.(Arg124His) + p.(Ile247Asn). (B) The assay was performed in triplicate (average of triplicate measurements ± standard deviations). Results are expressed as ratio between each samples and WT. *, $p < 0.05$. (C) Immunoblot analysis of TGFBIp aggregates. TGFBIp samples were incubated for 1 h at 37 °C then immunoblotted with β-mercaptoethanol. Lane 1; WT; lane 2: WT + p.(His174Asp); lane 3: WT + p.(Ile247Asn); lane 4: WT; lane 5: p.(Arg124His); lane 6: p.(Arg124His) + p.(His174Asp); lane 7: p.(Arg124His) + p.(Ile247Asn); lane 8: p.(Arg124His).

Supplementary Table S1. Primers Designed for Amplification of *TGFBI*.

Exon	Primers	Sequences (5'……..3')	Product size (bp)
1	Ex1 F	CGCTCTCACTTCCCTGGAG (19)	323
	Ex1 R	TTTTAGTTCGGGCTTTGTCC (20)	
2	Ex2 F	GGGAGTCATTAAAGTGGGGTGGA (23)	196
	Ex2 R	AGCTTGGTCTCCTGGCTGGTTAC (23)	
3	Ex3 F	CAACTTAGTGGAGAGGGGCCAGA (23)	206
	Ex3 R	CTCTCTCCCACCATTCCCTTCC (22)	
4	Ex4 F	GCCATCCCTCCTTCTGTCTTCTG (23)	217
	Ex4 R	CCGGGCAGACGGAGGTCATC (20)	
5	Ex5 F	ACTGACACCCTGTCCTTCCTCCT (23)	261
	Ex5 R	AGCCCACACATGGAACAGAAATG (23)	
6	Ex6 F	CTGCTCATCCTTGCTGCTTCTCT (23)	249
	Ex6 R	AGAGTTCCTGCTAGGCCCCTCTT (23)	
7	Ex7 F	TCTGTGGGGAGTGCCAGAGTC (21)	234
	Ex7 R	CAAATGAGGCAGCAGCAGGA (20)	
8	Ex8 F	TGGACCCTGACTTGACCTGAGTC (23)	311
	Ex8 R	AAAGGATGGCAGAAGAGATGGTG (23)	
9	Ex9 F	CCCTGGGGTGGATGAATGATAAA (23)	251
	Ex9 R	GCCTCCAGGGACAATCTAACAGG (23)	
10	Ex10 F	ATTGCAGGAGCACATCTCTCTGG (23)	230
	Ex10 R	GCTTCCCAGGAGCATGATTTAGG (23)	
11	Ex11 F	GCCCCTCGTGGAAGTATAACCAG (23)	248
	Ex11 R	ATCCCACTCCAGCATGACCACT (22)	
12	Ex12 F	TGACAGGTGACATTTTCTGTGTGTG (25)	224
	Ex12 R	GGGCCCTGAGGGATCACTACTTT (23)	
13A	Ex13A F	TGACCAGGCTAATTACCATTCTTGG (25)	210
	Ex13A R	CAGCCTTTGATTTGCAGGACACT (23)	
13B	Ex13B F	CTTGACCAGGCTATTTACCATTCTT (25)	281
	Ex13B R	GGTTGTGTGTGTATAATTCCATCC (24)	
14	Ex14 F	TGCTCTACTTTCAACCACTACTCTG (25)	198
	Ex14 R	CCAACTGCCACATGAAGAAAAGG (23)	
15	Ex15 F	TCACTCTGGTCAAACCTGCCTTT (23)	183
	Ex15 R	CCTCTATGGCCCAAACAGAGGAC (23)	
16	Ex16 F	GCCATTGTCATAAGCAGTTGCAG (23)	176
	Ex16 R	ATACAGCAGATGGCAGGCTTGG (22)	
17	Ex17 F	TGGGGAGATCTGCACCTATTTGA (23)	710
	Ex17 R	GGTCAGCACACTGTACCATGCAC (23)	

Supplementary Table S2. Previous reports of double mutations of the *TGFBI* gene in the literature review.

Mutation 1	Mutation 2	Location	Phenotype	Ethnicity	Authors
**p.(Arg124Leu)**	**p.(Thr125_Glu126del)**	Cis	Atypical severe GCD	French	Dighiero *et al* (2000)
**p.(Arg124Cys)**	**p.(Gly470*)**	Trans	Severe LCD1	Japanese	Sakimoto *et al* (2003)
**p.(Ala546Asp)**	**p.(Pro551Gln)**	ND but likely Cis	LCD1	African American	Klintworth *et al* (2004)
**p.(Ala546Asp)**	**p.(Pro551Gln)**	ND but likely Cis	Atypical LCD1/3A	African American	Aldave *et al* (2004)
**p.(Ala549Thr)**	**p.(Arg555Trp)**	Trans	Severe GCD1	German	Frising *et al* (2006)
**p.(Arg124His)**	**p.(Asn544Ser)**	Trans	GCD2 + LCD	Japanese	Yamada *et al* (2009)
**p.(Leu550Pro)**	**p.(His626Arg)**	ND	Atypical GCD	Mexican	Zenteno *et al* (2009)
**p.(Arg514Pro)**	**p.(Phe515Leu)**	Cis	Severe LCD	Chinese	Zhong *et al* (2010)
**p.(Met502Val)**	**p.(Arg555Gln)**	Cis	Atypical TBCD	French	Niel-Butschi *et al* (2011)
**p.(Arg124His)**	**p.(Ser104Lysfs*27)**	ND but likely Trans	Severe GCD2	Chinese	Yam *et al* (2012)
**p.(Arg124His)**	**p.(Ala179*)**	Trans	Severe GCD2	Korean	Song *et al* (2015)
**p.(Pro501Thr)**	**p.(Arg555Gln)**	Cis	TBCD	Korean	Chae *et al* (2016)
**p.(Val113Ile)**	**p.(Leu558Pro)**	Trans	Atypical LCD	Spanish	Ann *et al* (2017)

Abbreviations are as follows: GCD, granular corneal dystrophy; LCD, lattice corneal dystrophy; ND, not determined; TBCD, Thiel-Behnke corneal dystrophy.
